# Distinct behavioral phenotypes in ethanol-induced place preference are associated with different extinction and reinstatement but not behavioral sensitization responses

**DOI:** 10.3389/fnbeh.2014.00267

**Published:** 2014-08-08

**Authors:** João V. N. Pildervasser, Karina P. Abrahao, Maria L. O. Souza-Formigoni

**Affiliations:** ^1^Unidade de Dependência de Drogas, Departament of Psicobiologia, Universidade Federal de Sao PauloSao Paulo, Brazil; ^2^Laboratory of Integrative Neuroscience, National Institute of Alcohol Abuse and Alcoholism, National Institutes of HealthRockville, MD, USA

**Keywords:** behavioral sensitization, CPP, different phenotypes, ethanol, extinction, individual variability

## Abstract

Conditioned place preference (CPP) is a model to study the role of drug conditioning properties. In outbred strains, individual variability may affect some behavioral measures. However, there are few studies focusing on understanding how different phenotypes of ethanol conditioned behavior may influence its extinction, reinstatement, and behavioral adaptation measures. We used male Swiss Webster mice to study different phenotypes related to ethanol conditioning strength, reinstatement and behavioral sensitization. Mice went through a CPP procedure with ethanol (2.2 g/kg, i.p.). After that, one group of mice was submitted to repeated extinction sessions, while another group remained in their home cages without any drug treatment. Mice went through environmental and ethanol priming (1.0 g/kg, i.p.) reinstatement tests. Ethanol priming test reinstated the conditioned behavior only in the animals kept in the home-cage during the abstinence period. Besides, the ethanol conditioned behavior strength was positively correlated with the time required to be extinguished. In the second set of experiments, some mice went through a CPP protocol followed by behavioral sensitization (five i.p. administrations of ethanol 2.2 g/kg or saline per week, for 3 weeks) and another group of mice went through sensitization followed by CPP. No positive correlation was observed between ethanol CPP strength and the intensity of behavioral sensitization. Considering that different phenotypes observed in CPP strength predicted the variability in other CPP measures, we developed a statistics-based method to classify mice according to CPP strength to be used in the evaluation of ethanol conditioning properties.

## Introduction

Ethanol abuse is a serious problem which afflicts society and has already been related to 3.8% of all deaths worldwide (Rehm et al., 2009; Laramee et al., 2013). Its chronic exposure induces modifications in brain functioning from molecular to synaptic levels and modifies behavior which may facilitate dependence development (Cannady et al., 2013; Luchtmann et al., 2013; Young et al., 2014). Some major obstacles faced during ethanol dependence treatment are craving and relapse (George et al., 2012). Stressful situations, drug priming and context re-exposure are known to work as triggers for craving which may lead to relapse (Shalev et al., 2002; Bossert et al., 2005; George and Koob, 2011).

Several experimental paradigms are used to evaluate drug rewarding effect, craving, relapse and environmental cues [for review see Sanchis-Segura and Spanagel (2006) and Martin-Fardon and Weiss (2013)] such as the conditioned place preference (CPP) (Cunningham et al., 2006). Through a classical Pavlovian conditioning, it is possible to evaluate the capacity of ethanol rewarding effect to induce changes in the conditioned behavior (Tzschentke, 1998, 2007). The CPP paradigm allows the study of environment-evoked relapse based on the animals' approach to the conditioned environment after a period of abstinence (Liu et al., 2008; Aguilar et al., 2009) and it has been used to investigate and manipulate drugs of abuse-related memories (Monfils et al., 2009; Font and Cunningham, 2012; Groblewski and Cunningham, 2012; Xue et al., 2012).

It is important to consider that not all animals exposed to a drug of abuse will necessarily develop dependence (Deroche-Gamonet et al., 2004). A key question that remains unanswered is why this occurs. One possibility is the fact that there is a significant individual variability in the behavioral and neurobiological adaptations induced by chronic exposure to drugs. Some studies focused on ethanol-induced behavioral sensitization individual variability (Souza-Formigoni et al., 1999; Abrahao et al., 2013; Nona et al., 2014), a form of drug-dependent behavioral plasticity associated with addiction vulnerability, but very few investigated it using the CPP paradigm. Even though Tesone-Coelho et al. (2013) reported different phenotypes in the ethanol-induced CPP, they did not study its influence on other related behaviors such as extinction and reinstatement.

Furthermore, Steketee and Kalivas (2011) demonstrated that there is a significant overlap between the neurochemical circuitries of sensitization and reinstated drug-seeking behavior. In addition, the authors suggest that sensitization of the neural reward circuitry as posed by Robinson and Berridge (2003) is a factor associated with the reinstated behavior which has been experimentally demonstrated by Keiflin et al. (2008). The authors showed that prior administration of cocaine, right before food delivery in operant conditioning sessions, induces behavioral sensitization. Moreover, after an extinction procedure, cocaine administration reinstated the operant conditioned behavior (Keiflin et al., 2008). This finding suggests that cocaine acquired a discriminative property which may be potentiated due to the sensitization. In a recent work, Yamamoto et al. (2013) showed that rats classified as high or low responders to the acute effect of cocaine presented different phenotypes regarding behavioral sensitization and CPP. These authors demonstrated that the animals classified as low responders developed, concomitantly, behavioral sensitization and CPP to cocaine (Allen et al., 2007; Yamamoto et al., 2013).

The present study investigated the relationship between the different phenotypes in ethanol-induced conditioned preference strength and the extinction of this conditioned behavior. We hypothesized that mice previously conditioned in the CPP and exposed to an extinction protocol would not have their preference behavior reinstated in the same magnitude presented by mice left in their home cage during the abstinence period. We also believe that ethanol conditioned behavior strength should be positively correlated with the time required for extinction and the reinstatement conditioned behavior strength. We also investigated the relationship between the different phenotypes in the CPP and behavioral sensitization models in order to assess whether the phenotypes would be expressed together or not.

## Materials and methods

### Subjects

Two hundred and ninety seven male Swiss Albino mice from the colony of CEDEME (Centro de Desenvolvimento de Modelos Experimentais para Medicina e Biologia—Universidade Federal de Sao Paulo) were housed in plastic cages (30 cm x 19 cm x 13 cm) in groups of 4 or 5 animals and given free access to food and water. They were kept in a temperature-controlled colony room (22 ± 1°C) with lights on from 07:00 AM to 07:00 PM. Mice were approximately 90 days old at the beginning of each experiment. All procedures were performed in accordance with the National Institutes of Health (NIH) Principles of Laboratory Animal Care (1985) and approved by the Committee of Ethics in Research of the Universidade Federal de Sao Paulo (CEP# 0036/12). All experiments were performed between 08:00 AM and 01:00 PM.

### Behavioral protocols

#### Conditioned place preference

The CPP apparatus (Insight Ltda., Sao Paulo, Brazil) consisted of an acrylic and stainless steel box (14 × 44 cm × 15 cm) divided into three compartments: two cue-compartments containing visual cues, either horizontal or vertical black and white stripe walls, and tactile cues, either mesh or bar floor, and one central compartment considered neutral that had gray walls and smooth floor. The cues combinations were: horizontal stripes/bar floor and vertical stripes/mesh floor. Each compartment was separated from the neutral one by guillotine doors and presented infrared beams used to obtain the spatial location of the animals throughout the tests.

The CPP protocol consisted of three phases: habituation (1st day), conditioning (2nd to 9th day) and post-conditioning test (10th day). Habituation: This phase was designed to evaluate drug-free baseline preference for the compartments. Animals were placed in the neutral compartment and had free access to the whole apparatus for 15 min. Animals that spent 65% or more of the total test time in one of the cue-compartments were excluded from the experiment in order to maintain an unbiased protocol. There were no differences in baseline cue-compartment preference between the saline and ethanol groups before the conditioning phase [*t*_(297)_ = 0.71, *p* > 0.05]. Conditioning: in this phase, mice from the ethanol group received four intraperitoneal (i.p.) ethanol (2.2 g/kg, 15% w/v) and four i.p. saline (0.9% w/v NaCl) injections on alternate days, while mice in the saline group received saline every day. After each administration, mice were confined to one of the cue-compartments to which they had been randomly assigned. Half of the animals received ethanol in the horizontal stripes/bar floor compartment and half received it in the vertical stripes/mesh floor compartment. Animals were kept in the cue-compartment (CS+ = conditioned compartment or CS− = non-conditioned-compartment) for 15 min with the guillotine doors closed. Post-conditioning test: 24 h after the last conditioning day, mice went through the post-conditioning test which was similar to the habituation phase. They were placed into the neutral compartment and had free access to the whole apparatus for 15 min in a drug-free condition.

Since the time spent in the central compartment does not seem to influence the development of the CPP (see Supplementary Material Table 1S) the preference for the CS+ compartment was measured by the preference delta—an index obtained by the percentage of total test time spent in the CS+ compartment minus the percentage of total test time spent in the CS− compartment. This index was calculated in each test allowing us to compare the preference levels in the habituation test with those from other tests (Cunningham et al., 2003).

#### Behavioral sensitization

The behavioral sensitization protocol and the sensitization classification method were similar to those described in previous studies (Abrahao et al., 2012, 2013; Abrahao and Souza-Formigoni, 2012). On the first day, in order to assess the horizontal locomotor activity in a novel environment, all the animals were initially evaluated in a 15-min drug-free situation session in Opto-Varimex cages (Columbus Instruments, Ohio, USA; 47.5 × 25.7 × 20.5 cm), which detected locomotor activity by the interruption of horizontal photoelectric beams. There were no differences in baseline activity between the saline and ethanol treatment groups [*t*_(117)_ = −1.36, *p* > 0.05]. From the day after the baseline test on, mice were given i.p. injections of either saline (0.9% w/v NaCl) or ethanol (2.2 g/kg, 15% w/v) five times per week for 3 weeks. Mice went through locomotor activity tests on the first drug administration and then once a week (tests 1–4). On the test days, mice received saline or ethanol and were immediately placed in the Opto-Varimex cages for 15 min. According to their locomotor response on the 4th test, the ethanol-treated mice were sorted and classified as “sensitized” (those whose activity was in the upper 33% of the locomotor activity distribution) or “non-sensitized” (those whose activity was in the lower 33% of the locomotor activity distribution). This kind of methodology has been used in many studies to detect groups with extreme profiles of locomotor response after a chronic drug treatment, allowing us to evaluate possible factors associated with the individual variability.

### Experiments

#### Experiment 1: ethanol CPP protocol in Swiss Webster mice

In order to evaluate if the proposed CPP protocol would induce conditioning in our animals, we conducted a first experiment with 31 mice conditioned with ethanol (*n* = 22) or saline (*n* = 9).

#### Experiment 2: CPP, extinction and reinstatement

Sixty seven mice were conditioned with ethanol (*n* = 47) or saline (*n* = 20) in the CPP procedure and submitted to an “extinction” protocol. Five animals were excluded from this group for not meeting the inclusion criterion mentioned above. Extinction protocol: 24 h after the post-conditioning phase, all animals went through the 14 extinction tests. According to Muller and de Wit (2011), repeated tests with free access to the whole apparatus and saline pairings with the previously drug-associated compartment procedures are effective in reducing the conditioned preference and preventing reinstatement; however, as stated by the same authors, only the repeated testing procedure is able to yield information about extinction across the days. Since the main focus of the present work was to evaluate possible influences of the individual variability on the CPP procedure, each extinction test was similar to the habituation and post-conditioning test in which mice were placed in the neutral compartment, in a drug-free condition, and had free access to the whole apparatus for 15 min. If an animal presented levels of preference delta in the 95% confidence interval of the habituation levels of the ethanol-conditioned group for at least 2 days in a row, the first of these days was used to compute the number of days needed for the conditioning to be extinguished. The last day of this phase was named “environmental reinstatement” test. Twenty four hours after this test, all animals went through the “ethanol reinstatement” test. Mice received a “priming” dose of ethanol (1.0 g/kg, 15% w/v) and were immediately placed in the central compartment of the CPP. Animals had free access to the whole apparatus for 15 min.

In the “no extinction” protocol, 85 animals were conditioned with ethanol (*n* = 61) or saline (*n* = 24) in the CPP procedure. Four animals were excluded for not meeting the inclusion criterion. No extinction protocol: 24 h after the post-conditioning phase, all animals returned to their home-cages where they were kept without drug treatment or exposure to the CPP apparatus for 13 days. On the 14th day, mice went through the environmental reinstatement test and, 24 h later, to ethanol reinstatement test.

#### Experiment 3: relationship between CPP and behavioral sensitization to ethanol

***Experiment 3A: CPP followed by behavioral sensitization.*** Thirty two mice received ethanol (*n* = 24) or saline (*n* = 8) during the CPP procedure. One animal was excluded from this experiment for not meeting the inclusion criterion. After the post-conditioning test, mice remained in the home cage without any drug treatment for 14 days. After that period all mice went through a behavioral sensitization protocol. Mice were allocated to either ethanol (*n* = 23) or saline (*n* = 9) treatment for the behavioral sensitization procedure and then classified according to their locomotor activity.

***Experiment 3B: behavioral sensitization followed by CPP.*** Eighty two mice received ethanol (*n* = 61) or saline (*n* = 21) during the behavioral sensitization procedure. After test 4, mice were classified according to their locomotor activity and then remained in the home cage in a drug free situation for 14 days. After that period they went through the CPP procedure. Seven animals were excluded from this experiment for not meeting the inclusion criterion. In order to provide balanced groups for the CPP procedure, mice were allocated to saline or ethanol treatment according to their behavioral sensitization classification.

### Data analysis

We used the Two-Way Analysis of Variance (ANOVA) for repeated measures for the CPP data analysis. In all CPP experiments the independent factor was group. The first group was composed by animals that received saline in both compartments (saline-conditioned) and the second group by mice that received ethanol in one compartment and saline in the other compartment (ethanol-conditioned). The dependent variable (preference delta) was evaluated at two moments: in the habituation and the post-conditioning tests, generating the second independent factor (test). In Experiment 2, a second repeated measures ANOVA was conducted in which the two groups were ethanol-conditioned mice submitted to the extinction protocol or to the no extinction protocol. The preference delta was evaluated in four occasions: habituation, post-conditioning test, environmental and ethanol reinstatement tests. In Experiments 3A and 3B, One-Way ANOVAs were conducted considering saline, non-sensitized and sensitized as the levels of factor group and the preference delta in the post-conditioning test as the dependent variable. For the behavioral sensitization data, the locomotor activity levels were analyzed by repeated measures ANOVA, considering group (saline, sensitized and non-sensitized mice) as the independent factor. Newman-Keuls tests for multiple comparisons were used for *post-hoc* analysis when the ANOVA detected a significant effect. Although the novelty-exposure test results are presented in the figures, these data were not included in the statistical analysis.

Pearson's correlation tests were used in Experiments 2 and 3. In Experiment 2, regarding the extinction protocol, correlation analysis was used to measure the association between the preference delta in the post-conditioning test and the number of days required to return to the 95% confidence interval of the habituation levels in the ethanol-conditioned group. A similar analysis was conducted between the preference delta in the post-conditioning test and in the ethanol reinstatement test. In the no extinction protocol, we tested the correlation between the preference delta in the post-conditioning test and in the environment (or ethanol) reinstatement tests. In Experiments 3A and 3B, we evaluated the association between the preference delta in the post-conditioning test and the locomotor activity levels in test 4, only in animals treated with ethanol in both procedures. The level of significance was set to 5% in all analyses.

#### Cluster analysis and ROC curve prediction

Data from the ethanol-conditioned mice regarding Experiments 1–3A were taken together (*n* = 154) to investigate the different phenotypes related to ethanol reinforcing properties. K-means hierarchical cluster analysis considering the preference delta in the post-conditioning test as dependent variable was set to generate three groups. Exploratory linear discriminant analysis considering the clusters as groups and the preference delta as the dependent variables was conducted to characterize each cluster. Through a ROC (Receiver Operating Characteristic) curve analysis we determined the cut-off points for each cluster. We conducted another set of analysis using the same procedures with preference score instead of preference delta as the dependent variable, given that both measures are equally used to analyze the conditioned behavior in the CPP procedure (Cunningham et al., 2003).

To further investigate the individual variability in the CPP procedure, we analyzed the data from Experiment 2 using the classification generated by the model. We decided to use only the classification based on the preference delta given that this measure was able to assort the animals in more discrete clusters.

Statistical analyses were made in SPSS 18.0 (IBM) and Statistica 12 (Statsoft).

## Results

### Experiment 1: ethanol CPP protocol in Swiss Webster mice

**Figure 1:**
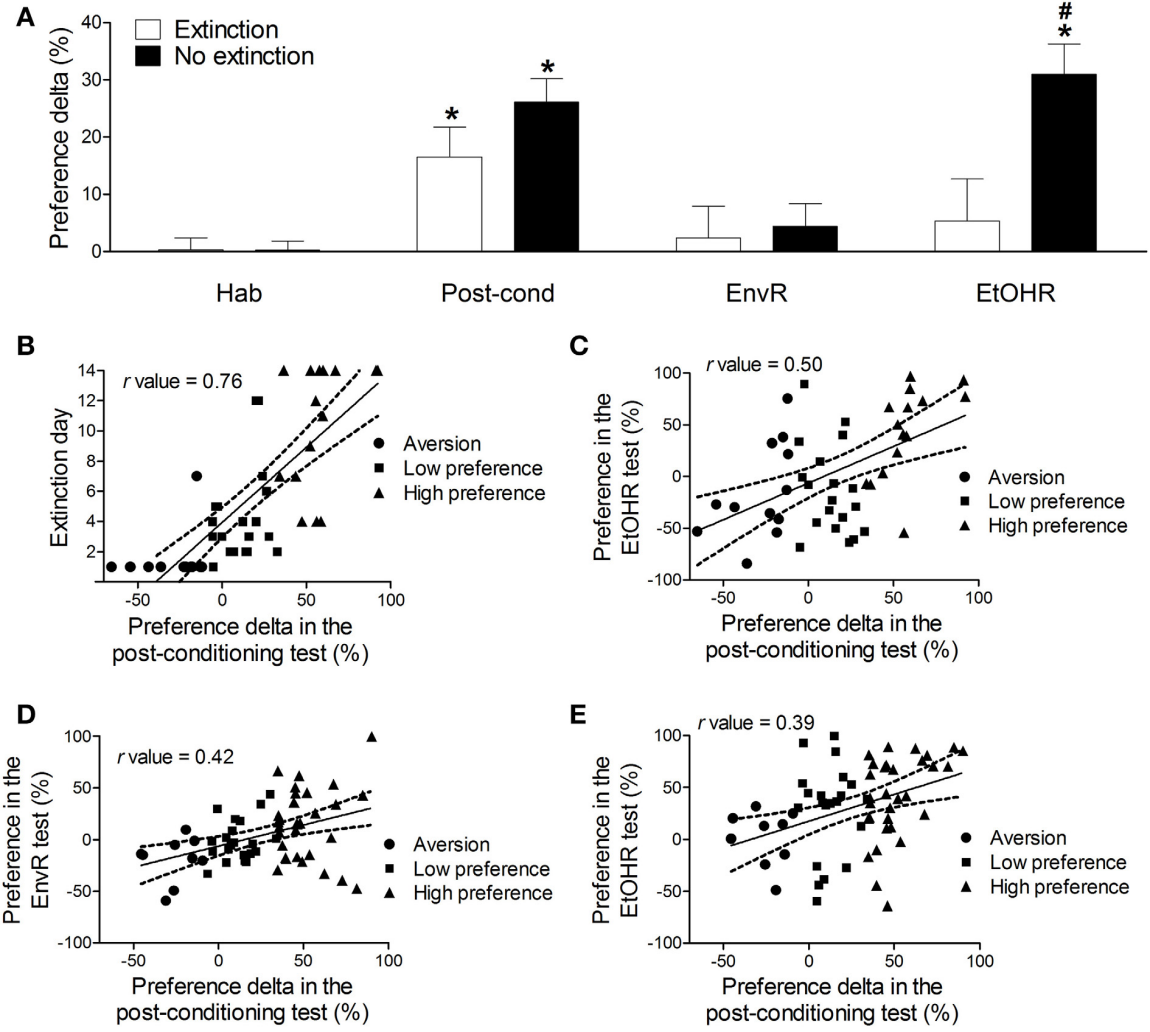
**(A)** Preference delta for the CS+ compartment (mean ± s.e.m) of mice conditioned with 2.2 g/kg ethanol from Experiment 2 extinction protocol (*n* = 47) and no extinction protocol (*n* = 61) in the habituation (Hab), post-conditioning test (Post-cond), environmental reinstatement (EnvR) and ethanol “priming” reinstatement (EtOHR). Preference delta was obtained through the percentage of total test time spent in the CS+ compartment minus the percentage of total test time spent in the CS- compartment in each test. ^*^Significantly higher preference delta than in habituation test (*P* < 0.05). #Significantly higher preference than the extinction group in the same test (*P* < 0.001). **(B)** Pearson's correlation (*r* = 0.76, *P* < 0.05) between the preference delta in the post-conditioning test and the number of days required to return to the 95% confidence interval of preference delta habituation levels in the ethanol-conditioned mice (extinction protocol). **(C)** Pearson's correlation (*r* = 0.50, *P* < 0.05) between the preference delta in the post-conditioning test and the preference delta in the ethanol reinstatement test of ethanol-conditioned mice (extinction protocol). **(D)** Pearson's correlation (*r* = 0.42, *P* < 0.05) between the preference delta in the post-conditioning test and the preference delta in the environment reinstatement test in the ethanol-conditioned mice (no extinction protocol). **(E)** Pearson's correlation (*r* = 0.39, *P* < 0.05) between the preference delta in the post-conditioning test and the preference delta in the ethanol reinstatement test in ethanol-conditioned mice (no extinction protocol). Each point represents a single animal classified according to its preference for the CS+ compartment, determined by the classification model described in the text (See Table 1).

To evaluate whether the ethanol-conditioned mice developed a place preference for the CS+ compartment, a repeated measures ANOVA was conducted considering group (saline- or ethanol-conditioned animals) as the independent factor and tests (preference delta in the habituation and post-conditioning test) as the dependent variable. The ANOVA identified significant effects of test [*F*_(1, 29)_ = 3.86, *P* = 0.05] and interaction between group and test factors [*F*_(1, 29)_ = 3.90, *P* = 0.05], but not of group factor [*F*_(1, 29)_ = 2.22]. *Post-hoc* analysis detected that in the post-conditioning test the preference delta levels of ethanol-conditioned mice were higher than the levels of saline-conditioned mice and also than their own levels in the habituation (*P* < 0.05) (Figure 1S). This result confirms that the proposed CPP protocol establishes a clear preference for the ethanol paired compartment.

### Experiment 2: CPP, extinction and reinstatement

**Figure 2:**
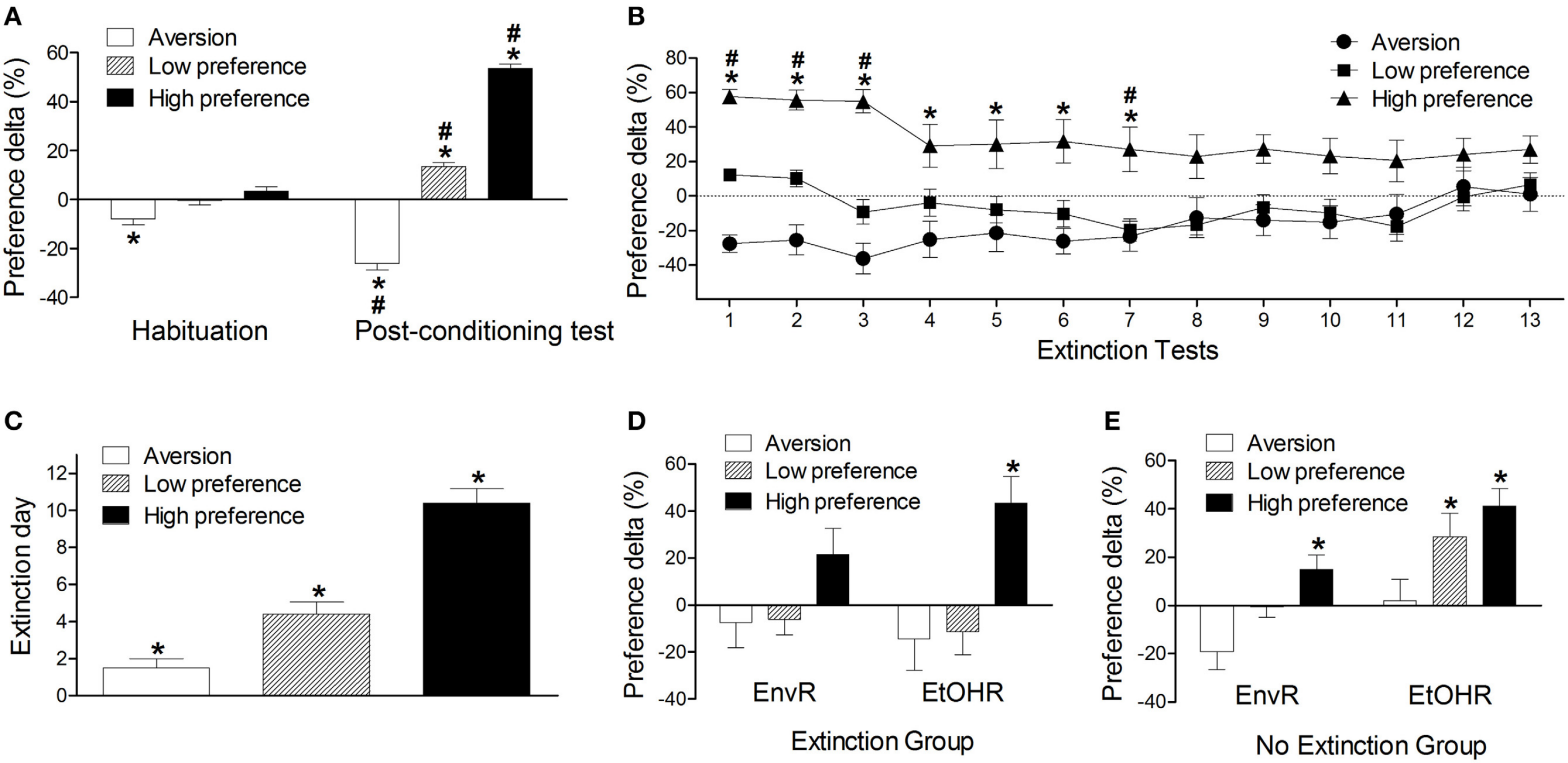
**(A)** Preference delta for the CS+ compartment (mean ± s.e.m) of mice conditioned with 2.2 g/kg ethanol from Experiments 1–3A in the habituation and post-conditioning test classified as aversion (*n* = 32), low preference (*n* = 57) or high preference (*n* = 65) according to the classification model based on the preference delta. ^*^Differs from the other groups in the same test. #Differs from the habituation test (*P* < 0.001). **(B)** Preference delta for the CS+ compartment in the extinction tests phase of ethanol-conditioned mice (Experiment 2 extinction protocol) classified as aversion (*n* = 12), low preference (*n* = 20) and high preference (*n* = 15). ^*^Differs from the aversion group in the same test (*P* < 0. 05). #Differs from the low preference group in the same test (*P* < 0. 05). **(C)** Number of days required for the conditioned behavior to be extinguished (mean ± s.e.m) of ethanol-conditioned mice (Experiment 2 extinction protocol) classified as aversion, low preference and high preference. ^*^Differs from other groups (*P* < 0.01). **(D)** Preference delta for the CS+ compartment of ethanol-conditioned mice in the environmental (EnvR) and ethanol reinstatement (EtOHR) tests of ethanol-conditioned mice (Experiment 2 extinction protocol) classified as aversion, low preference and high preference. *Higher than other groups in the same test (*P* < 0.001). **(E)** Preference delta for the CS+ compartment in the environmental and ethanol reinstatement tests of ethanol-conditioned mice (Experiment 2 no extinction protocol) classified as aversion (*n* = 9), low preference (*n* = 21) and high preference (*n* = 31). *Differs from the aversion group in the same test (*P* < 0.01). Not all significant differences between tests are depicted in this figure. See text for details.

For the extinction protocol, a repeated measures ANOVA considering group (saline- or ethanol-conditioned animals) as the independent factor detected a significant effect of interaction between group and tests factors [*F*_(1, 65)_ = 6.49, *P* < 0.05]. We also observed a trend regarding the group factor [*F*_(1, 65)_ = 3.50, *P* = 0.06] but no effect of the factor test [*F*_(1, 65)_ = 1.97]. Although no differences were observed between ethanol and saline groups in the habituation test, in the post-conditioning test ethanol-conditioned mice spent more time in the CS+ compartment than the saline group as well as than their own levels in the habituation (*P* < 0.05). Similar results were observed regarding the no extinction protocol. In this case, the ANOVA detected significant effects of group [*F*_(1, 83)_ = 11.17, *P* < 0.01], tests [*F*_(1, 83)_ = 7.31, *P* < 0.01] and interaction between group and test factors [*F*_(1, 83)_ = 16.39, *P* < 0.001] (Figure 2S).

In order to compare the preference delta during the habituation, post-conditioning, environmental and ethanol “priming” reinstatement tests, we conducted a repeated measures ANOVA, with ethanol-conditioned mice from both protocols (extinction and no extinction) as the group factor (Figure 1A). Significant effects of group [*F*_(1, 106)_ = 4.86, *P* < 0.05], tests [*F*_(3, 318)_ = 13.19, *P* < 0.001] and interaction between group and tests factors [*F*_(3, 318)_ = 4.05, *P* < 0.01] were observed. In both groups, a *post-hoc* analysis detected significantly higher preference for the CS+ compartment in the post-conditioning test than in the habituation (*p* < 0.05). No differences were observed among habituation, environmental and ethanol priming reinstatement tests in the extinction group (Figure 1A). Thus, there was no reinstatement of conditioned behavior after the extinction tests. On the other hand, in the no extinction group we observed higher levels of preference for the CS+ compartment in the ethanol reinstatement test than in the habituation and in the environmental reinstatement tests (*P* < 0.001), at similar levels to those from the post-conditioning test (Figure 1A). In this case, the priming dose of ethanol was able to reinstate the conditioned behavior. Indeed, in the ethanol priming reinstatement test animals from the no extinction group had a significantly higher preference than those from the extinction group (*P* < 0.001). The extinction protocol prevented the ethanol-induced reinstatement behavior in those mice repeatedly exposed to the CPP apparatus, but not in those left in their home cages during the withdrawal period.

Figure 1 also shows the correlations between preference delta in the post-conditioning test and extinction day (B); preference in the ethanol reinstatement test from the extinction protocol (C) and the no extinction protocol (E); preference in the environmental reinstatement test in the no extinction protocol (D). Each point represents a single animal classified according to its preference for the CS+ compartment, determined by the classification model which will be described in the next section.

As shown in Figure 1B, the Pearson's correlation test found a strong positive correlation (*r* = 0.76, *P* < 0.05) between the preference delta levels in the post-conditioning test and the number of days required to extinction (return to the 95% confidence interval of the habituation levels of preference delta), indicating that a higher level of conditioning requires more time to be extinguished. Figure 1C shows a moderate positive correlation (*r* = 0.50, *P* < 0.05) between the preference delta in the post-conditioning test and in the ethanol priming reinstatement test (EtOHR), suggesting that those mice with stronger ethanol conditioning may be prone to reinstate the conditioned preference even after the extinction tests.

Considering the no extinction group, a moderate positive correlation was found between the preference delta levels in the post-conditioning test and in the environment reinstatement test (*r* = 0.42, *P* < 0.05, Figure 1D) and also between the preference delta levels in the post-conditioning test and in the ethanol priming reinstatement test (*r* = 0.39, *P* < 0.05, Figure 1E).

### Classification model for CPP

**Figure 3:**
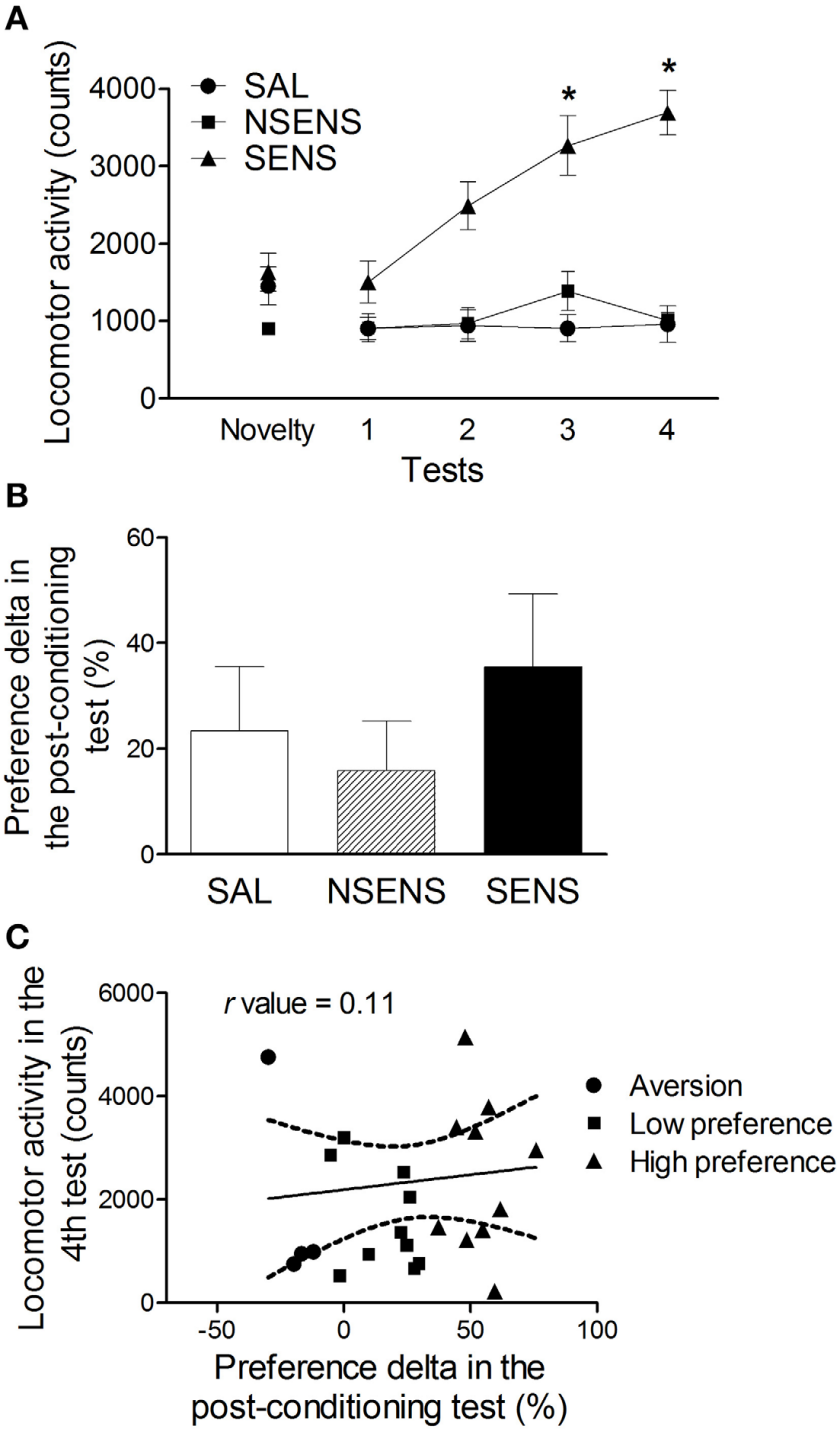
**(A)** Locomotor activity (mean ± s.e.m) for 15 min of mice treated with saline (SAL) (*n* = 9) or 2.2 g/kg ethanol (NSENS = 8; SENS = 8) in the novelty-exposure test and in tests 1–4 (Experiment 3A). Based on their activity in test 4, the ethanol-treated mice were classified as “sensitized” or “non-sensitized.” ^*^Higher than SAL and NSENS groups on the same test (*P* < 0.05) and higher than their own levels in test 1 (*P* < 0.05). **(B)** Preference delta for the CS+ compartment (mean ± s.e.m) in the post-conditioning test of ethanol-conditioned mice (Experiment 3A) classified as saline (SAL), “non sensitized” (NSENS) and “sensitized” (SENS) in the behavioral sensitization protocol. **(C)** Pearson's correlation (*r* = 0.11, *P* > 0.05) between the preference delta in the post-conditioning test and the locomotor activity on test 4 of ethanol-treated mice. Each point represents a single animal classified according to its preference for the CS+ compartment, determined by the classification model described in the text (See Table 1).

**Figure 4:**
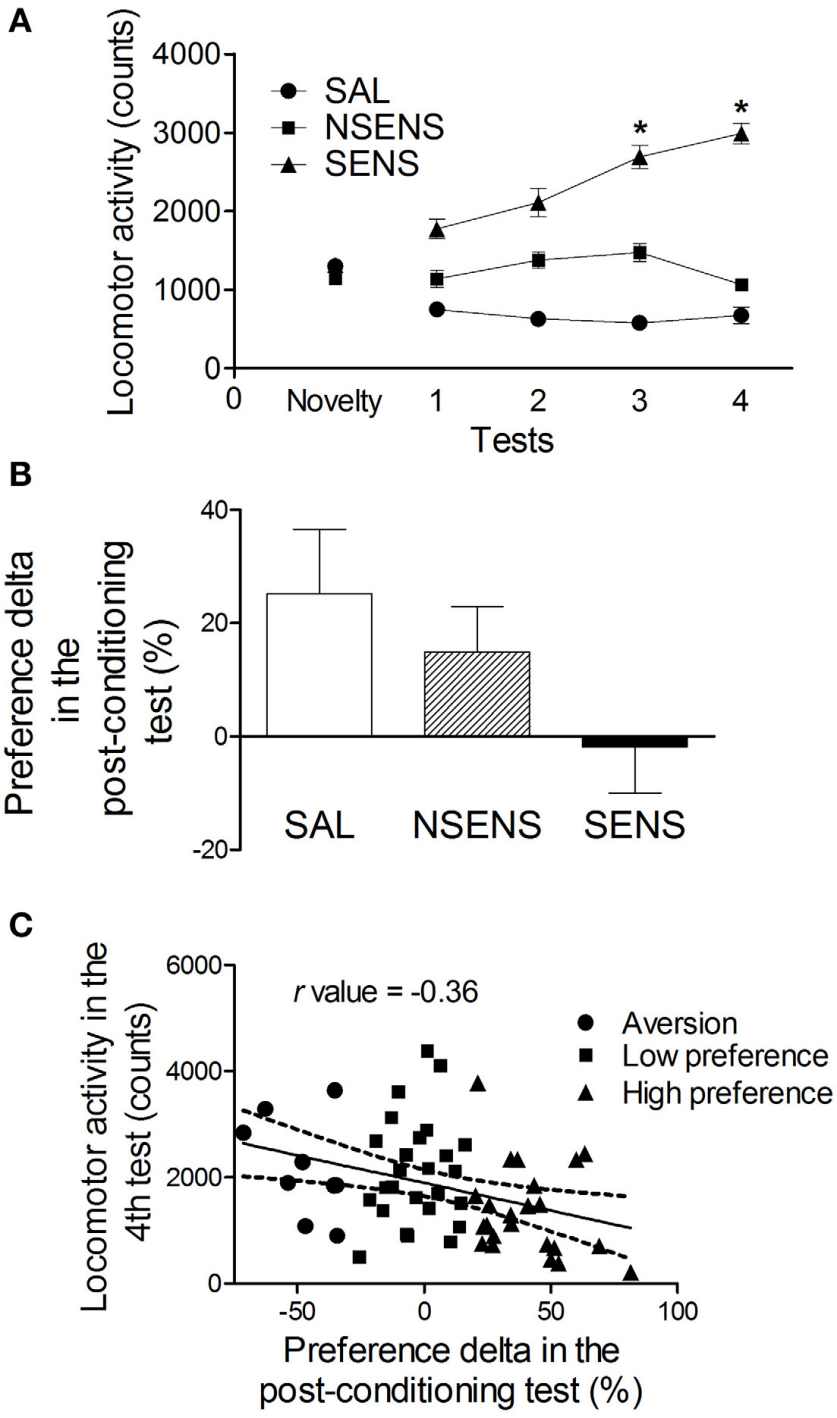
**(A)** Locomotor activity (mean ± s.e.m) for 15 min of mice treated with saline (SAL) (*n* = 21) or 2.2 g/kg ethanol (NSENS = 19; SENS = 22) in the novelty-exposure test and in tests 1–4 (Experiment 3B). Based on their activity in test 4, the ethanol-treated mice were classified as “sensitized” or “non-sensitized.” *Higher than SAL and NSENS groups on the same test (*P* < 0.05) and higher than their own levels in test 1 (*P* < 0.05). **(B)** Preference delta for the CS+ compartment (mean ± s.e.m) in the post-conditioning test of ethanol-conditioned mice (Experiment 3B) classified as SAL (*n* = 9), NSENS (*n* = 16) and SENS (*n* = 17) in the behavioral sensitization protocol. **(C)** Pearson's correlation (*r* = −0.36, *P* < 0.05) between the preference delta in the post-conditioning test and the locomotor activity in test 4 of ethanol-treated mice. Each point represents a single animal classified according to its preference for the CS+ compartment, determined by the classification model described in the text (See Table 1).

Considering the high levels of variability regarding the conditioned behavior phenotype to ethanol in the CPP and its correlation with the time to be extinguished, as well as with the conditioned preference in the reinstatement tests, we developed a classification model for the CPP using a statistics-based approach. A cluster analysis classified the ethanol-conditioned mice into three groups, interpreted by us as: aversion, low preference and high preference. A linear discriminant analysis confirmed the difference among clusters (Wilks' Lambda = 0.16, *F*_(2, 151)_ = 373.201, *P* < 0.001). In order to evaluate the accuracy of cluster classification, a ROC curve analyses was performed for each cluster. The optimal cut-off points, sensitivity, 1—specificity and the percentage of animals classified in each cluster are presented in Table 1 (See also Figure 3S). The AUC (area under the curve) for each extreme cluster in both analyses was 1.0. The analyses with the preference score model are available as Supplementary Material (Table 2S, Figures 3S, 4S).

**Table 1:** **Cut-off points for each cluster (based on preference delta) along with the respective sensitivity and 1-specificity values, generated by ROC curve analysis**.

	**Cut-off points**	**Sensitivity**	**1-Specificity**	**Ethanol group**	**Saline group**
Aversion	*x* < −8%	0.97	0.0	20.8% (*n* = 32)	44.2% (*n* = 27)
Low preference	−8% ≤ preference delta < 34%	–	–	37.0% (*n* = 57)	49.2% (*n* = 30)
High preference	preference delta ≥ 34%	0.98	0.0	42.2% (*n* = 65)	6.6% (*n* = 4)

The percentage of animals classified in each cluster in the saline- and ethanol-conditioned groups is also presented.

We ran complementary statistical analyses with the CPP variables considering the classification model proposed above (see Figure 2). Repeated measures ANOVA with groups (aversion, low preference and high preference) as the independent factor detected significant effects of group [*F*_(2, 151)_ = 221.49, *P* < 0.001], tests [*F*_(1, 151)_ = 89.11, *P* < 0.001] and interaction between group and tests factors [*F*_(1, 151)_ = 144.91, *P* < 0.001]. While the preference delta in the post-conditioning test of the aversion group was lower than in the habituation, both low and high preference groups' preference deltas were higher in the post-conditioning test than their own levels in the habituation (*P* < 0.001). In the post-conditioning test all groups were significantly different from each other: while the aversion group presented a negative preference, the high preference group presented the highest preference (*P* < 0.001) (Figure 2A). These results indicate that the classification method was effective to differentiate the three groups according to their ethanol conditioned behavior.

Considering the extinction time course among the aversion, low and high preference groups, significant effects of group [*F*_(2, 44)_ = 19.88, *P* < 0.001], tests [*F*_(13, 572)_ = 2.29, *P* < 0.01] and interaction between group and tests factors [*F*_(26, 572)_ = 2.45, *P* < 0.001] were detected (Figure 2B). The ANOVA did not detect significant differences between the low preference and the aversion groups across the extinction tests. On the other hand, the preference delta levels of the high preference group were higher than those presented by the aversion group from the 1st extinction test to the 7th test (*P* < 0.05). The high preference group also had a higher preference delta than the low preference group from the 1st to the 3rd test and in the 7th test (*P* < 0.05) (Figure 2B). Differences among tests were observed in the aversion group only between the 3rd and the 12th test (*P* < 0.05). In the low preference group, no differences were observed across the extinction tests. Regarding the high preference group, the preference levels in the 1st, 2nd, and 3rd extinction tests were significantly higher than in the 5th, 8th, 10–12th, and 14th tests (*P* = 0.05) (Figure 2B). Figure 2C shows the mean of days necessary for the extinction to be established. A One-Way ANOVA considering the extinction day as the dependent variable identified a significant effect of the group factor [*F*_(2, 44)_ = 28.07, *P* < 0.001]. All groups differed from each other. The aversion group needed fewer days to extinct the conditioned behavior and the high preference group more days (*P* < 0.05) (Figure 2C).

When we evaluated the preference delta in the environmental and in the ethanol reinstatement tests, the ANOVA detected significant effects of group [*F*_(2, 44)_ = 12.06, *P* < 0.001], but not of tests [*F*_(1, 44)_ = 0.13], or interaction between group and tests factors [*F*_(2, 44)_ = 1.08]. However, an unprotected *post-hoc* test identified that the high preference group presented higher preference for the CS+ compartment than the other two groups in the ethanol reinstatement test (*P* < 0.01) (Figure 2D). A similar analysis was conducted with data from those animals that were left in the home cage during the withdrawal period (no extinction group). In this case, the ANOVA detected significant effects of group [*F*_(2, 58)_ = 7.75, *P* < 0.01] and tests [*F*_(1, 58)_ = 13.11, *P* < 0.001] but no interaction between group and test factors [*F*_(2, 58)_ = 0.08, *P* > 0.05]. An unprotected *post-hoc* analysis showed that animals from the high preference group presented higher levels of preference delta than the aversion group in the environmental reinstatement test (*P* < 0.001). Besides, both low and high preference groups presented higher preference delta than the aversion group in the ethanol reinstatement test (*P* < 0.01) (Figure 2E). Therefore, through this novel classification we identified three phenotypes of animals which have particular responses to ethanol conditioning, extinction and reinstatement behavior.

### Experiment 3A: CPP followed by behavioral sensitization

In a similar way as described before, those mice which received ethanol presented an increased preference for the CS+ compartment after the conditioning phase (*P* < 0.01), confirming the development of CPP for ethanol. Repeated measures ANOVA for the CPP test detected significant effects of tests [*F*_(1, 30)_ = 6.95, *P* < 0.05] and interaction between group and tests factors [*F*_(1, 30)_ = 4.27, *P* < 0.05] but not of group factor [*F*_(1, 30)_ = 3.23]. Ethanol-conditioned mice spent more time in the CS+ compartment in the post-conditioning test than the saline-conditioned mice (*P* < 0.05) (data not shown).

The same group of mice went through a behavioral sensitization protocol 14 days after the post-conditioning test (Figure 3A). As previously described, mice were classified according to their locomotor activity on the last day of the sensitization protocol. Repeated measures ANOVA considering group (saline, non-sensitized and sensitized animals) as the independent factor detected significant effects of group [*F*_(2, 22)_ = 25.25, *P* < 0.001], tests [*F*_(3, 66)_ = 18.29, *P* < 0.001] and interaction between group and tests factors [*F*_(6, 66)_ = 12.57, *P* < 0.001]. In test 4, the sensitized group presented significantly higher activity than the saline-treated animals (*P* < 0.01) and then their own levels in tests 1–3 (*P* < 0.05). Non-sensitized mice, presented similar activity levels as those observed in the saline group. Thus, mice classified as sensitized showed robust behavioral sensitization with progressive increase in the activity counts during ethanol treatment (*P* < 0.05), while the non-sensitized group did not. It is important to notice that there were no differences in baseline or acute (test 1) locomotor activity levels among saline, sensitized and non-sensitized groups.

Considering the classification of animals based on their behavioral sensitization performance, a new statistical analysis was conducted with the CPP data. No differences were found in the preference delta of the post-conditioning test among saline, non-sensitized and sensitized groups [One-Way ANOVA: *F*_(2, 17)_ = 0.70] (Figure 3B). Besides, no significant correlation was found between the preference delta for the CS+ compartment in the post-conditioning test and the locomotor activity levels in test 4 (*r* = 0.11 *P* > 0.05) (Figure 3C), suggesting that these two behavioral phenotypes (high levels of conditioning and of sensitization) are not expressed in the same animals.

### Experiment 3B: behavioral sensitization followed by CPP

Figure 4A shows the development of behavioral sensitization to the stimulant effect of ethanol. Similarly to the previous experiment, repeated measures ANOVA detected significant effects of group [*F*_(2, 59)_ = 102.58, *P* < 0.001], tests [*F*_(3, 177)_ = 11.64, *P* < 0.001] and interaction between group and tests factors [*F*_(6, 177)_ = 16.56, *P* < 0.001]. Sensitized mice presented a progressive increase in locomotor activity (*P* < 0.05) which was higher in test 4, than their own levels in tests 1–3 (*P* < 0.05) and then the one presented by the saline-treated group in the same tests (*P* < 0.001). Sensitized mice also presented higher activity than saline animals in the first locomotor test (*P* < 0.05) indicating an acute stimulant effect of ethanol in this group. Non-sensitized mice presented higher locomotor activity in tests 2 and 3 than saline-treated animals (*P* < 0.05).

In contrast with the previous CPP experiments, ethanol-conditioned mice preference for the CS+ compartment did not increase after the conditioning phase. Considering ethanol or saline treatment as the grouping variable, a repeated measures ANOVA did not detect significant effects of group [*F*_(1, 80)_ = 2.53, *P* > 0.05], tests [*F*_(1, 80)_ = 0.68, *P* > 0.05] or interaction between group and tests [*F*_(1, 80)_ = 0.73, *P* > 0.05]. Thus, no conditioning was established (data not shown). Moreover, using the classification based on the behavioral sensitization performance as the independent factor, a One-Way ANOVA did not detect a significant influence of it on the ethanol-conditioned mice preference levels in the post-conditioning test [*F*_(2, 39)_ = 2.20, *P* = 0.12] (Figure 4B). However, it is important to note that, in the ethanol-conditioned group, there was a moderate negative correlation (*r* = −0.36, *P* < 0.05) between the preference delta in the post-conditioning test and the locomotor activity in test 4 (Figure 4C). These data suggest that the increased locomotor activity observed in the sensitized mice impaired the development of an ethanol conditioned behavior phenotype in the CPP protocol.

## Discussion

An important challenge about drug addiction is to understand why not every individual develops the phenotype of addiction after exposure to drugs of abuse (Piazza and Le Moal, 1996). Thus, not surprisingly, scientists have been giving increased attention to the development of adequate strategies to study this variability (Masur et al., 1986; Piazza and Le Moal, 1996; Deroche-Gamonet et al., 2004). In the present study, we identified distinct phenotypes in ethanol-induced place preference and its relationship with its extinction, reinstatement and behavioral sensitization. We developed a model to classify animals treated with ethanol regarding their conditioned behavior. In summary, we observed that ethanol conditioning strength in CPP was positively correlated with the time required for the conditioned behavior to be extinguished, as well as with the preference level in the ethanol reinstatement test. Furthermore, no positive correlation was observed between their behavioral profiles in the CPP and behavioral sensitization.

Since reinstatement of the conditioned behavior after a withdrawal period represents a major obstacle for dependence treatment (George and Koob, 2011; George et al., 2012), several studies have focused on the extinction of memories related to drug-cue association (Paolone et al., 2009; Xue et al., 2012; Poltyrev and Yaka, 2013). Although there is no consensus about the optimal approach to this matter, our results point to an important influence of the level of the conditioned behavior on its extinction. While for 25.5% of the animals few exposures (less than 7 sessions) without ethanol were enough to extinguish the ethanol conditioned behavior, 14.9% of them still preferred the CS+ compartment even after 14 days of daily exposure.

We have also shown that, after a priming dose of ethanol, animals from the extinction group had similar preference levels for the CS+ compartment as those observed in the habituation, while the animals from the no extinction group did not. Xue et al. (2012) showed that repeated exposure in a drug-free situation to cues previously paired with cocaine or heroin in a CPP procedure is not sufficient to prevent drug-priming reinstatement, being necessary the inclusion of a specific memory retrieval-extinction procedure. In light of the present results, the memory-retrieval extinction does not seem to be necessary to prevent ethanol-priming reinstatement in the CPP paradigm.

Behavioral individual variability to drug effects is an important characteristic of dependence that has been observed both in humans and animal models of addiction (Piazza and Le Moal, 1996; Deroche-Gamonet et al., 2004). As previously mentioned, we detected a significant variability in the CPP paradigm which is in accordance with a recent study (Tesone-Coelho et al., 2013). Considering that we observed an association between the conditioning strength behavior and other behavioral parameters of CPP such as reinstatement, we propose two novel methods to classify ethanol-induced preference based on the ethanol conditioned behavior in CPP. Using the delta preference values model, we clearly identified three different phenotypes of animals. Among the ethanol-conditioned mice some developed a more intense conditioning (high preference group) than others (low preference group), while another subset of animals developed place avoidance (aversion group). Subsequent analyses detected that animals from the low preference and aversion group required fewer days of extinction test to return to habituation levels of preference, those from the high preference group required a high number of tests or did not extinguish the conditioned behavior to ethanol.

It is important to note that, animals classified as high preference presented a higher preference delta than the other two groups in the ethanol reinstatement test even in the animals that went through the extinctions tests, suggesting a masking effect of the analysis of the whole group. This finding indicates that even after a 14-day period of extinction tests some animals still presented the conditioned behavior. Furthermore, in the ethanol reinstatement test, both low and high preference groups presented a higher preference than those from the aversion group. Thus, animals with a high preference phenotype present behavioral responses related to an increased susceptibility to relapse.

Contrary to our initial hypothesis, when the behavioral sensitization protocol preceded the CPP, sensitized mice did not express a higher place preference. One plausible explanation for this outcome is that chronic exposure to ethanol, followed by a withdrawal period, increased brain reward threshold (Schulteis et al., 1995; George et al., 2012) which was not reached during the CPP procedure. Also, this explanation is in line with previous findings from our group which showed that those animals classified as sensitized had higher levels of voluntary ethanol consumption after a similar withdrawal period as used in the present work (Abrahao et al., 2013). Based on these studies, we may infer that the sensitized animals would require an extended exposure to ethanol or an increased ethanol dose to experience the rewarding effects of ethanol in order to develop the conditioned behavior.

Despite this inference, another interpretation of the present result is possible. A recent work highlighted different neurobiological functioning for cocaine behavioral sensitization and CPP (Bocklisch et al., 2013). The authors demonstrated that high frequency stimulation of accumbal medium spiny neurons, which indirectly regulate the dopaminergic neurons in the ventral tegmental area (VTA) through GABA interneurons in the VTA, can increase the development of behavioral sensitization to cocaine and impair the development of cocaine CPP (Bocklisch et al., 2013). It is possible that the development of sensitization to ethanol elicited neurobiological adaptations that could negatively affect CPP. In addition, it is also possible to consider that since the sensitized animals still express behavioral sensitization after an administration of ethanol even following an 18-day withdrawal period (Abrahao et al., 2011, 2012), the increased locomotor activity during the ethanol conditioning pairings sessions may have disrupted the attentional control required for the establishment of the conditioned behavior (Byers and Serences, 2012).

Taken together, the present results emphasize the importance of investigating distinct phenotypes in behavioral models of addiction, as well as the relationship among them. Interestingly, the intensity of conditioning, observed in the post-conditioning test of CPP, was correlated to the time necessary for it to be extinguished. Besides, the extinction tests prevented the ethanol priming reinstatement of the conditioned behavior. These data strengthen the body of findings which point out that memory extinction procedures are beneficial for the treatment of craving and relapse to ethanol addiction (Lee et al., 2007; Torregrossa and Taylor, 2013). Finally, the non-contingent chronic exposure to ethanol and the development of behavioral sensitization impaired the CPP behavior. Further studies are necessary to investigate the neurobiological substrates underlying the negative correlation between behavioral sensitization development and CPP as well as to identify possible features associated with susceptibility to relapse. We believe that our CPP classification model should be applied to new cohorts of behaviorally characterized animals.

### Conflict of interest statement

The authors declare that the research was conducted in the absence of any commercial or financial relationships that could be construed as a potential conflict of interest.
