# Supplementary material for: Distinct behavioral phenotypes in ethanol-induced place preference are associated with different extinction and reinstatement but not behavioral sensitization responses
Source: Front Behav Neurosci. 2014 Aug 8;8:267. doi: 10.3389/fnbeh.2014.00267 (PMC4126182; doi:10.3389/fnbeh.2014.00267)
Supplement: Supplementary file 1 [file DataSheet1.DOCX]

***Supplementary Material***

**Distinct behavioral phenotypes in ethanol-induced place preference are associated with different extinction, reinstatement and behavioral sensitization responses**

**João Victor Nicodemos Pildervasser^1^, Karina Possa Abrahao^2^, Maria Lucia Oliveira Souza-Formigoni*^1^**

^1^ Departamento de Psicobiologia, Universidade Federal de São Paulo, São Paulo, Brazil

^2^ Laboratory of Integrative Neuroscience, NIAAA, NIH, Rockville, MD, USA

*** Correspondence:** Maria Lucia O. Souza Formigoni, Ph.D., Departamento de Psicobiologia, Universidade Federal de São Paulo (UNIFESP),Rua Napoleão de Barros, 950 - Sao Paulo, SP, 04024-002, Brazil
mlosformigoni@unifesp.br

1. **Supplementary Figures and Tables**


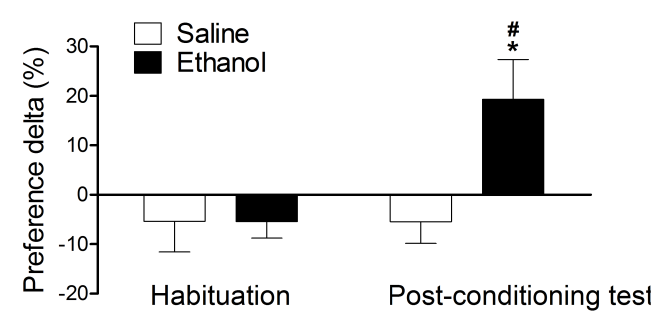


**Supplementary Figure 1S** Preference delta for the CS+ compartment (mean ± S.E.M) of mice conditioned with 2.2 g/kg ethanol (*n* = 22) or saline (*n* = 9) from experiment 1 in the habituation and post-conditioning test.

* higher preference delta than in habituation test (*P* < 0.05).

# higher preference than the saline group in the same test (*P* < 0.05).


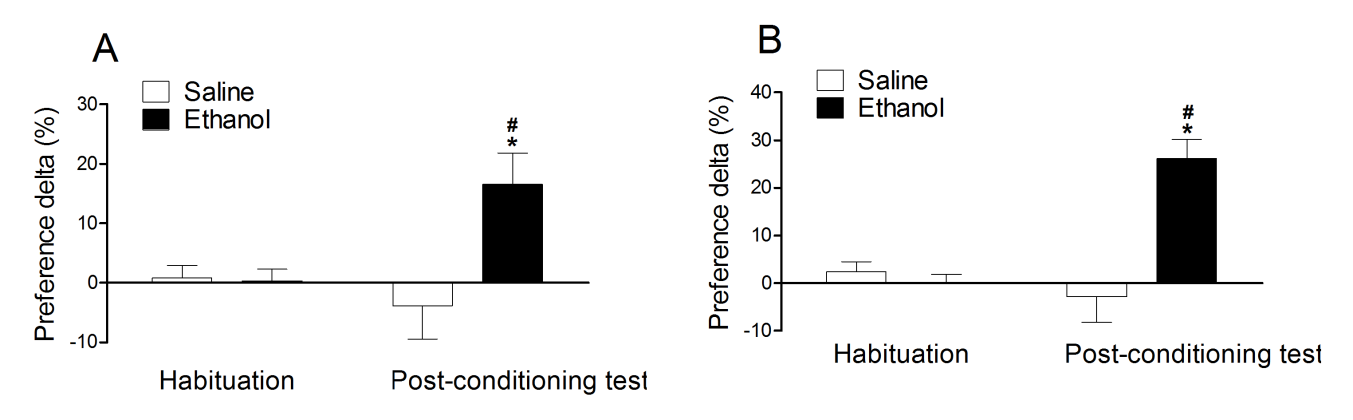


**Supplementary Figure 2S. (A)** Preference delta for the CS+ compartment (mean ± S.E.M) of mice conditioned with 2.2 g/kg ethanol (*n* = 47) or saline (*n* = 20) from Experiment 2 extinction protocol in the habituation and post-conditioning test. * higher preference delta than in habituation test (*P* < 0.05). # higher preference than the saline group in the same test (*P* < 0.05). **(B)** Preference delta for the CS+ compartment (mean ± S.E.M) of mice conditioned with 2.2 g/kg ethanol (*n* = 61) or saline (*n* = 24) from Experiment 2 no extinction protocol in the habituation and post-conditioning test. * higher preference delta than in habituation test (*P* < 0.05). # s higher preference than the saline group in the same test (*P* < 0.05).

**Supplementary Table 1S**. Cut-off points for each cluster (based on preference score) along with the respective sensitivity and 1- specificity values, generated by ROC curve analysis. The percentage of animals classified in each cluster in the saline- and ethanol-conditioned groups is also presented.

|  | Cutoff point | Sensitivity | 1-Specificity | Ethanol group | Saline group |
| --- | --- | --- | --- | --- | --- |
| Aversion | x < - 4% | .97 | 0.0 | 27.3% (*n* = 42) | 49.2% (*n* = 30) |
| Low preference | - 4% ≤ preference score < 26% | - | - | 42.2% (*n* = 65) | 47.5% (*n* = 29) |
| High preference | preference score ≥ 26% | .98 | 0.0 | 30.5% (*n* = 47) | 3.3% (*n* = 2) |


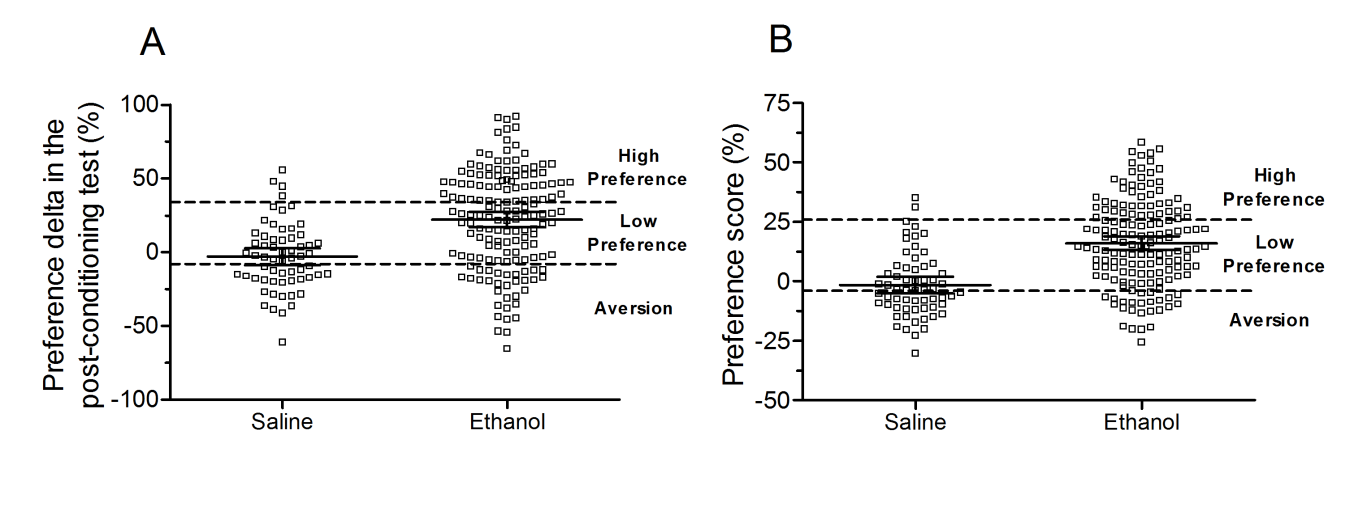

**Supplementary Figure 3S**. **(A)** Distribution of the preference delta of mice conditioned with 2.2 g/kg ethanol or saline from experiments 1, 2 and 3A in the post-conditioning test. Each dot represents an animal. The dotted lines represent the cutoff point used to separate each group (aversion, low preference and high preference) (See table 1). The continuous lines represent the mean and confidence interval 95%. **(B)** Distribution of the preference score of mice conditioned with 2.2 g/kg ethanol or saline from experiments 1, 2 and 3A. Each dot represents an animal. The dotted lines represent the cutoff point used to separate each group (aversion, low preference and high preference) (See table 1S). The continuous lines represent the mean and confidence interval 95%.


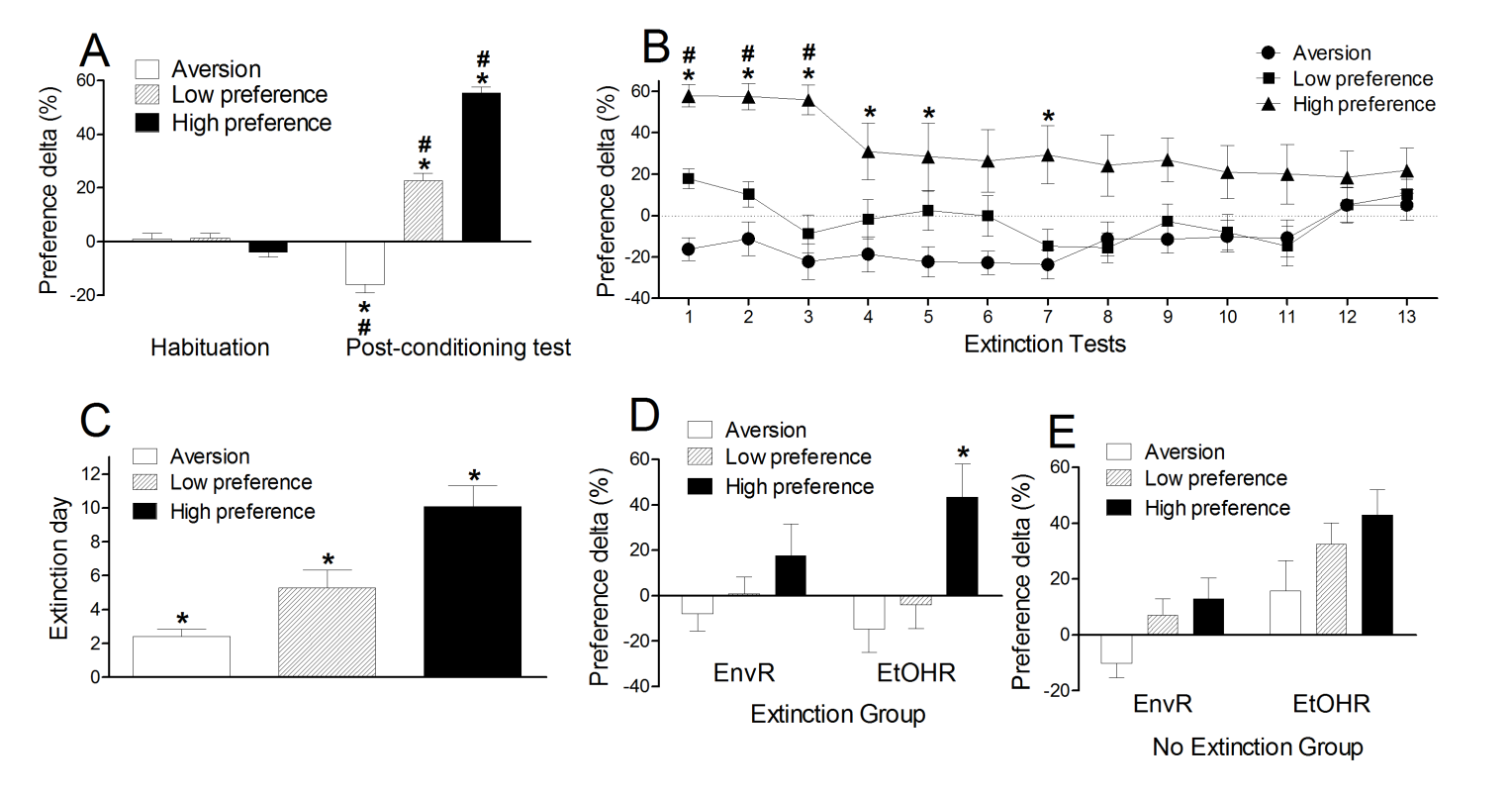


**Supplementary Figure 4S**. **(A)** Preference delta for the CS+ compartment (mean ± S.E.M) of mice conditioned with 2.2 g/kg ethanol from experiments 1, 2 and 3A in the habituation and post-conditioning test classified as aversion (*n* = 42), low preference (*n* = 65) or high preference (*n* = 47) according to the classification model based on the preference score. * Differs from the other groups in the same test (*P* < 0.001). # Differs from the habituation test (*P* < 0.001). **(B)** Preference delta for the CS+ compartment in the extinction tests phase of ethanol-conditioned mice (experiment 2 extinction protocol) classified as aversion (*n* = 17), low preference (*n* = 17) and high preference (*n* = 13). * Differs from the aversion group in the same test (*P* < 0. 05). # Differs from the low preference group in the same test (*P* < 0. 05). **(C)** Number of days required for the conditioned behavior to be extinguished (mean ± S.E.M) of ethanol-conditioned mice (experiment 2 extinction protocol) classified as aversion, low preference and high preference. * differs from other groups (*P* < 0.01). **(D)** Preference delta for the CS+ compartment of ethanol-conditioned mice in the environmental (EnvR) and ethanol reinstatement (EtOHR) tests of ethanol-conditioned mice (experiment 2 extinction protocol) classified as aversion, low preference and high preference. * higher than other groups in the same test (*P* < 0.05). **(E)** Preference delta for the CS+ compartment in the environmental and ethanol reinstatement tests of ethanol-conditioned mice (Experiment 2 no extinction protocol) classified as aversion (n = 17), low preference (n = 21) and high preference (n = 23). Each point represents a single animal classified according to its preference for the CS+ compartment, determined by the classification model described in the text (See Supplementary Table 1S).
